# Bis[bis­(4,4′-dimethyl-2,2′-bipyridine)(10,11,12,13-tetra­hydro­dipyrido[3,2-*a*:2′,3′-*c*]phenazine)ruthenium(II)] tetra­kis(perchlorate) acetonitrile disolvate monohydrate

**DOI:** 10.1107/S1600536810014005

**Published:** 2010-04-21

**Authors:** ChengHui Zeng, ZhengZheng Li, ZhenHua Liang, YunJun Liu, Fuhai Wu

**Affiliations:** a School of Pharmacy, Guangdong Pharmaceutical University, Guangzhou 510006, People’s Republic of China; b School of Public Health, Guangdong Pharmaceutical University, Guangzhou 510006, People’s Republic of China

## Abstract

The asymmetric unit of the title compound, [Ru(C_12_H_12_N_2_)_2_(C_18_H_14_N_4_)]_2_(ClO_4_)_4_·2CH_3_CN·H_2_O, contains two Ru^II^ complex cations, four perchlorate counter-anions, two uncoord­inated acetonitrile mol­ecules and one water mol­ecule. The Ru^II^ ions are chelated by one 10,11,12,13-tetra­hydro­dipyrido[3,2-*a*:2′,3′-*c*]phenazine (dpqc) and two 4,4′-dimethyl-2,2′-bipyridine (dmb) ligands in a distorted octa­hedral geometry. The uncoordinated water mol­ecule is disordered over three positions, with occupancy factors of 0.398 (9), 0.312 (8) and 0.290 (8). A supra­molecular structure is formed by weak π–π inter­actions between neighbouring mol­ecules, with face-to-face distances of 3.51 (1) Å [centroid–centroid distance 3.81 (1) Å].

## Related literature

For information on octa­hedral Ru^II^ polypyridyl complexes, see: Juris *et al.* (1988 ▶); MacDonnell *et al.* (1999 ▶). For Ru^II^ complexes with other ligand systems, see: Liu *et al.* (2009 ▶); Pellegrini & Aldrich-Wright (2003 ▶). For the preparation of dipyro[3,2-*a*:2′,3′-*c*](10,11,12,13-tetra­hydro)phenazine, see: Dickeson & Summers (1970 ▶).
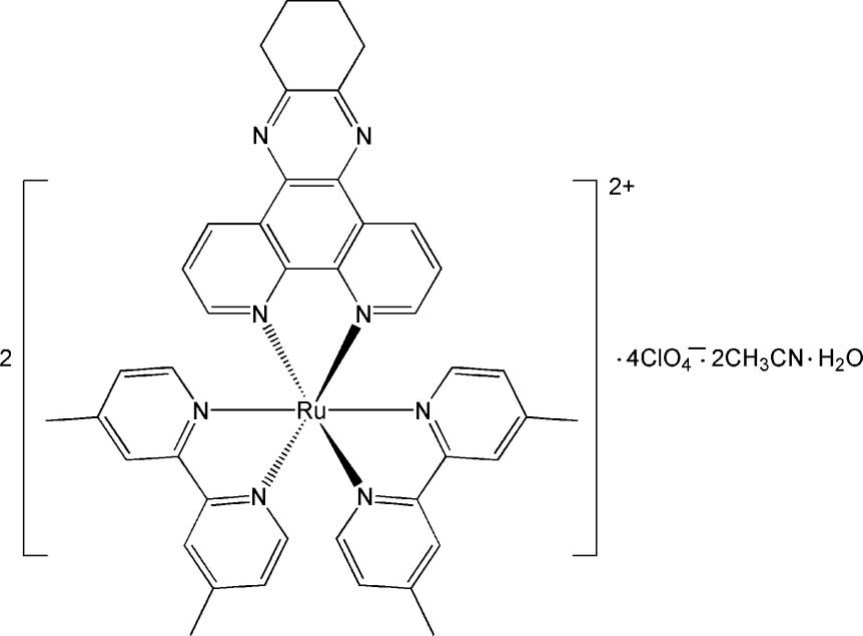


## Experimental

### 

#### Crystal data

[Ru(C_12_H_12_N_2_)_2_(C_18_H_14_N_4_)]_2_(ClO_4_)_4_·2C_2_H_3_N·H_2_O
*M* *_r_* = 2009.67
Triclinic, 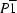

*a* = 12.8883 (15) Å
*b* = 15.2555 (18) Å
*c* = 22.659 (3) Å
α = 94.767 (2)°
β = 91.553 (3)°
γ = 94.068 (2)°
*V* = 4426.1 (9) Å^3^
*Z* = 2
Mo *K*α radiation
μ = 0.54 mm^−1^
*T* = 110 K
0.28 × 0.24 × 0.22 mm

#### Data collection

Bruker SMART APEX CCD diffractometer
Absorption correction: multi-scan (*SADABS*; Bruker, 2000 ▶) *T*_min_ = 0.863, *T*_max_ = 0.890
33829 measured reflections
17010 independent reflections
13351 reflections with *I* > 2σ(*I*)
*R*_int_ = 0.031

#### Refinement

*R*[*F* ^2^ > 2σ(*F* ^2^)] = 0.053
*wR*(*F* ^2^) = 0.136
*S* = 1.04
17010 reflections
1193 parameters
1 restraint
H-atom parameters constrained
Δρ_max_ = 0.59 e Å^−3^
Δρ_min_ = −1.05 e Å^−3^

### 

Data collection: *SMART* (Bruker, 2000 ▶); cell refinement: *SAINT* (Bruker, 2000 ▶); data reduction: *SAINT*; program(s) used to solve structure: *SHELXTL* (Sheldrick, 2008 ▶); program(s) used to refine structure: *SHELXTL*; molecular graphics: *SHELXTL*; software used to prepare material for publication: *SHELXTL*.

## Supplementary Material

Crystal structure: contains datablocks global, I. DOI: 10.1107/S1600536810014005/bg2339sup1.cif

Structure factors: contains datablocks I. DOI: 10.1107/S1600536810014005/bg2339Isup2.hkl

Additional supplementary materials:  crystallographic information; 3D view; checkCIF report

## Figures and Tables

**Fig. 1.**
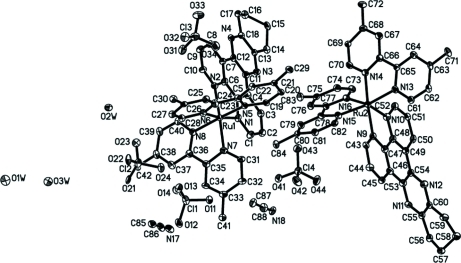
A view of the title compound showing the atom-numbering scheme and displacement ellipsoids drawn at 30% probability level, for the sake of clarity, the H atoms have been omitted.

**Fig. 2.**
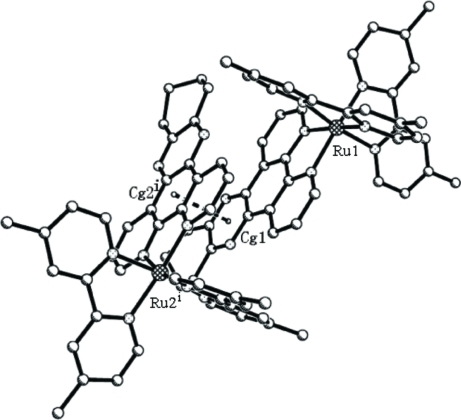
A view of the π–π interactions between neighbouring molecules. All H atoms have been omitted for clarity. ( i) 1+x,y,z, ii) -1+x,y,z ).

## supplementary crystallographic information

### Comment

Octahedral Ru^II^ polypyridyl complexes have aroused more and more interests of researchers because of their extensive applications in the fields of photochemistry, photophysics, photocatalysis, electrochemistry, biochemistry and so on (Juris *et al.*, 1988; MacDonnel *et al.*, 1999). Apart from the studies on ruthenium(II) complexes containing 2,2'-bipyridine (bpy) or 1,10-phenanthroline (phen) ligands, many other ligands system, such as 4,4'-dimethyl-2,2'-bipyridine (dmb) and 2,9-dimethyl-1,10-phenanthroline (dmp) have also been investigated (Liu *et al.*, 2009). Pellegrini has found that [Ru(dmb)_2_(dpqc)]^2+^ (where dpqc stands for dipyro[3,2-a:2',3'-c](10,11,12,13-tetrahydro)phenazine) has a great affinity with DNA (Pellegrini *et al.*, 2003).

In our attempts to obtain complexes with different structural properties, we present here the crystal structure of the title compound, 2[Ru(dmb)_2_(dpqc)].4(ClO_4_).2CH_3_CN.H_2_O (Fig. 1).

All the bond lengths and angles in the structure have normal values. There are two Ru^II^^2+^ complex cations in one asymmetric unit of the crystal structure, four perchlorate anions, two uncoordinated acetonitrile molecules and one water molecule. The Ru1(II) ion is in a distorted octahedral environment, coordinated by N5, N6, N7, and N8 from two 4,4'-dimethyl-2,2'-bipyridine (dmb) ligands, and N1 and N2 from one dipyro[3,2-a:2',3'-c](10,11,12,13-tetrahydro)phenazine(dpqc) ligand (Fig. 1). The same as Ru1(II), the Ru2(II) ion is also in a distorted octahedral environment, coordinated by N13, N14, N15, and N16 from two other dmb ligands, and N9 and N10 from the remaining dpqc ligand (Fig. 1). The uncoordinated water molecule is disordered over three different positions with site occupancy factors of 0.398 (9), 0.312 (8) and 0.290 (8), respectively. The Ru—N bond lengths range from 2.048 (3) to 2.081 (3) Å.

Weak π–π interactions occur between neighbouring molecules (Fig. 2), involving the C11, C12, N3, N4, C13 and C18 ring (centroid Cg1) and the C46, C47, C48, C49, C53 and C54 ring (centroid Cg2). ( Cg1···Cg2^ii^ interaction, (ii) 1+x, y, z : face to face distance 3.51 (1)Å, centroid-centroid distance 3.81 (1)Å, dihedral angle 3.68 (18) °).

### Experimental

Dipyro[3,2-a:2',3'-c](10,11,12,13-tetrahydro)phenazine (dpqc) was prepared by modified method reported in the literature (Dickeson *et al.*, 1970). A mixture of phenanthroline-5,6-diamine 0.21 g (1 mmol), 1,2-cyclohexanedione 0.112 g (1 mmol) and glacial acetic acid (30 cm^3^) was refluxed with stirring for 6 h, The cooled solution was diluted with water and neutralized with concentrated aqueous ammonia, a pale yellow-green precipitate was obtained. The product was recrystallized from methanol to give pale yellow-green needles. Yield: 91%. Anal. Calcd (%) for C_18_H_14_N_4_: C 75.51, H 4.93, N 19.57; Found (%): C 75.54, H 4.95, N 19.60%. FAB-MS: m/z = 287 [M+1].

The complex 2[Ru(dmb)_2_(dpqc)].4(ClO_4_).2CH_3_CN.H_2_O was synthesized by a modified method respect of Pellegrini, *et al.*, 2003. A mixture of cis-[Ru(dmb)_2_Cl_2_].2H_2_O (0.288 g, 0.5 mmol) and dpqc (0.161 g, 0.5 mmol) in EtOH (40 cm^3^) was refluxed under argon for 8 h to give a clear red solution. Upon cooling, a red precipitate was obtained by dropwise addition of saturated aqueous NaClO_4_ solution. The crude product was purified by column chromatography on neutral alumina with CH_3_CN-toluene (3:1, v/v) as eluent. The mainly brown red band was collected. The solvent was removed under reduced pressure and a red powder was obtained. Yield: 62%. Anal. Calcd (%) for C_44_H_42_N_9_Cl_2_O_8.5_Ru: C 52.59, H 4.21, N 12.55%. Found (%): C 52.57, H 4.22, N 12.56;

Red single crystals of the complex suitable for an X-ray crystallographic study was grown from acetonitrile and ethanol(v:v 1:1) at room temperature.

### Refinement

In the asymmetric unit there is one water molecule disordered into three different sites with occupancy factors of 0.398 (9), 0.312 (8) and 0.290 (8), respectively.

C-H's were positioned geometrically and allowed to ride, with C—H = 0.95 (CH), 0.99 (CH2) and 0.98 (CH3) Å and with Uiso(H) = 1.2 (1.5 for methyl)Ueq(C). H atoms of water molecules were determined based on difference Fourier maps and possible hydrogen bonding scheme and allowed to ride with Uiso(H) = 1.2Ueq(O). The highest residual electron density was found 0.42 Å from C4 and the deepest hole 0.76 Å from H69.

### Crystal data

**Table:**

[Ru(C_12_H_12_N_2_)_2_(C_18_H_14_N_4_)]_2_(ClO_4_)_4_·2C_2_H_3_N·H_2_O	*Z* = 2
*M**_r_* = 2009.67	*F*(000) = 2060
Triclinic, *P*1	*D*_x_ = 1.508 Mg m^−^^3^
Hall symbol: -P 1	Mo *K*α radiation, λ = 0.71073 Å
*a* = 12.8883 (15) Å	Cell parameters from 8166 reflections
*b* = 15.2555 (18) Å	θ = 2.2–26.6°
*c* = 22.659 (3) Å	µ = 0.54 mm^−^^1^
α = 94.767 (2)°	*T* = 110 K
β = 91.553 (3)°	Block, red
γ = 94.068 (2)°	0.28 × 0.24 × 0.22 mm
*V* = 4426.1 (9) Å^3^	

### Data collection

**Table:**

Bruker SMART APEX CCD diffractometer	17010 independent reflections
Radiation source: sealed tube	13351 reflections with *I* > 2σ(*I*)
graphite	*R*_int_ = 0.031
phi and ω scans	θ_max_ = 26.0°, θ_min_ = 1.3°
Absorption correction: multi-scan (*SADABS*; Bruker, 2000)	*h* = −15→15
*T*_min_ = 0.863, *T*_max_ = 0.890	*k* = −18→18
33829 measured reflections	*l* = −27→27

### Refinement

**Table:**

Refinement on *F*^2^	Primary atom site location: structure-invariant direct methods
Least-squares matrix: full	Secondary atom site location: difference Fourier map
*R*[*F*^2^ > 2σ(*F*^2^)] = 0.053	Hydrogen site location: inferred from neighbouring sites
*wR*(*F*^2^) = 0.136	H-atom parameters constrained
*S* = 1.04	*w* = 1/[σ^2^(*F*_o_^2^) + (0.08*P*)^2^ + 1.99*P*] where *P* = (*F*_o_^2^ + 2*F*_c_^2^)/3
17010 reflections	(Δ/σ)_max_ < 0.001
1193 parameters	Δρ_max_ = 0.59 e Å^−^^3^
1 restraint	Δρ_min_ = −1.04 e Å^−^^3^

### Special details

**Table:**

Geometry. All esds (except the esd in the dihedral angle between two l.s. planes) are estimated using the full covariance matrix. The cell esds are taken into account individually in the estimation of esds in distances, angles and torsion angles; correlations between esds in cell parameters are only used when they are defined by crystal symmetry. An approximate (isotropic) treatment of cell esds is used for estimating esds involving l.s. planes.Least-squares planes (x,y,z in crystal coordinates) and deviations from them (* indicates atom used to define plane)11.8722 (0.0072) x + 4.5367 (0.0201) y + 1.8609 (0.0347) z = 11.2527 (0.0245)* 0.0208 (0.0023) N5 * -0.0016 (0.0024) C19 * -0.0186 (0.0025) C20 * 0.0201 (0.0024) C21 * -0.0016 (0.0026) C22 * -0.0191 (0.0026) C23Rms deviation of fitted atoms = 0.016111.7218 (0.0080) x + 5.3416 (0.0209) y - 1.4418 (0.0359) z = 5.9766 (0.0285)Angle to previous plane (with approximate esd) = 8.71 ( 0.14 )* -0.0166 (0.0024) N15 * 0.0120 (0.0026) C78 * 0.0034 (0.0026) C79 * -0.0139 (0.0026) C80 * 0.0095 (0.0026) C81 * 0.0056 (0.0025) C82Rms deviation of fitted atoms = 0.0111-6.2753 (0.0160) x + 13.6394 (0.0094) y + 1.8591 (0.0302) z = 4.0544 (0.0360)Angle to previous plane (with approximate esd) = 85.12 ( 0.10 )* -0.0128 (0.0025) C11 * 0.0028 (0.0024) C12 * 0.0112 (0.0023) N3 * 0.0089 (0.0022) N4 * 0.0004 (0.0022) C13 * -0.0105 (0.0022) C18Rms deviation of fitted atoms = 0.0090-6.9702 (0.0165) x + 13.1801 (0.0117) y + 2.1159 (0.0348) z = 6.7539 (0.0375)Angle to previous plane (with approximate esd) = 3.68 ( 0.18 )* 0.0000 (0.0028) C46_$1 * 0.0000 (0.0027) C47_$1 * 0.0000 (0.0026) C48_$1 * 0.0000 (0.0026) C49_$1 * 0.0000 (0.0027) C53_$1 * 0.0000 (0.0027) C54_$1Rms deviation of fitted atoms = 0.0000
Refinement. Refinement of *F*^2^ against ALL reflections. The weighted *R*-factor wR and goodness of fit *S* are based on *F*^2^, conventional *R*-factors *R* are based on *F*, with *F* set to zero for negative *F*^2^. The threshold expression of *F*^2^ > σ(*F*^2^) is used only for calculating *R*-factors(gt) etc. and is not relevant to the choice of reflections for refinement. *R*-factors based on *F*^2^ are statistically about twice as large as those based on *F*, and *R*- factors based on ALL data will be even larger.In the asymmetric unit there are one disordered water molecules. They occupy three different positions and theirs site occupancy factors were refined with free variable and validated as theirs site occupancy factors are 0.398 (9), 0.312 (8) and 0.290 (8) for O1W, O2W and O3W, respectively.H atoms on C atoms were positioned geometrically and refined as riding atoms, with C—H = 0.95 (CH), 0.99 (CH_2_) and 0.98 (CH_3_) Å and with Uiso(H) = 1.2 (1.5 for methyl)Ueq(C). H atoms of water molecules were determined based on difference Fourier maps and possible hydrogen bonding scheme and refined as riding, with Uiso(H) = 1.2Ueq(O). The highest residual electron density was found 0.42 Å from C4 and the deepest hole 0.76 Å from H69.

### Fractional atomic coordinates and isotropic or equivalent isotropic displacement parameters (Å^2^)

**Table:**

	*x*	*y*	*z*	*U*_iso_*/*U*_eq_	Occ. (<1)
C1	0.6303 (3)	0.4699 (3)	0.84001 (17)	0.0318 (8)	
H1	0.5653	0.4411	0.8263	0.038*	
C2	0.6565 (3)	0.4762 (3)	0.90069 (17)	0.0312 (8)	
H2	0.6088	0.4534	0.9278	0.037*	
C3	0.7521 (3)	0.5159 (2)	0.92102 (17)	0.0284 (8)	
H3	0.7711	0.5184	0.9620	0.034*	
C4	0.8235 (3)	0.5536 (3)	0.87991 (17)	0.0289 (8)	
C5	0.7896 (3)	0.5455 (2)	0.82082 (17)	0.0287 (8)	
C6	0.8517 (3)	0.5810 (3)	0.77826 (17)	0.0286 (8)	
C7	0.9478 (3)	0.6246 (2)	0.79480 (16)	0.0248 (7)	
C8	1.0081 (3)	0.6632 (3)	0.74848 (17)	0.0305 (8)	
H8	1.0738	0.6946	0.7572	0.037*	
C9	0.9658 (3)	0.6521 (3)	0.69137 (17)	0.0312 (8)	
H9	1.0033	0.6759	0.6602	0.037*	
C10	0.8711 (3)	0.6077 (2)	0.67904 (18)	0.0302 (8)	
H10	0.8450	0.6015	0.6392	0.036*	
C11	0.9195 (3)	0.5972 (2)	0.89644 (17)	0.0293 (8)	
C12	0.9817 (3)	0.6327 (2)	0.85389 (16)	0.0266 (7)	
C13	1.0430 (3)	0.6450 (2)	0.96969 (15)	0.0208 (7)	
C14	1.0753 (3)	0.6540 (3)	1.03458 (17)	0.0302 (8)	
H14A	1.0891	0.5950	1.0468	0.036*	
H14B	1.0165	0.6750	1.0577	0.036*	
C15	1.1704 (3)	0.7162 (3)	1.05022 (17)	0.0321 (8)	
H15A	1.1995	0.7046	1.0895	0.038*	
H15B	1.1504	0.7778	1.0527	0.038*	
C16	1.2534 (3)	0.7044 (3)	1.00367 (17)	0.0317 (8)	
H16A	1.3154	0.7448	1.0155	0.038*	
H16B	1.2748	0.6432	1.0022	0.038*	
C17	1.2143 (3)	0.7230 (2)	0.94231 (17)	0.0260 (7)	
H17A	1.2126	0.7875	0.9406	0.031*	
H17B	1.2636	0.7016	0.9126	0.031*	
C18	1.1078 (3)	0.6799 (2)	0.92651 (16)	0.0236 (7)	
C19	0.5732 (3)	0.6681 (2)	0.76030 (16)	0.0258 (7)	
H19	0.5762	0.6441	0.7976	0.031*	
C20	0.5405 (3)	0.7509 (2)	0.75806 (16)	0.0264 (7)	
H20	0.5197	0.7826	0.7931	0.032*	
C21	0.5375 (3)	0.7895 (2)	0.70382 (16)	0.0254 (7)	
C22	0.5637 (3)	0.7370 (2)	0.65303 (16)	0.0283 (8)	
H22	0.5608	0.7595	0.6152	0.034*	
C23	0.5933 (3)	0.6535 (3)	0.65850 (17)	0.0312 (8)	
C24	0.6182 (3)	0.5926 (3)	0.60735 (18)	0.0332 (8)	
C25	0.6034 (3)	0.6126 (3)	0.54942 (17)	0.0305 (8)	
H25	0.5749	0.6662	0.5413	0.037*	
C26	0.6303 (3)	0.5541 (2)	0.50318 (15)	0.0226 (7)	
C27	0.6670 (3)	0.4753 (2)	0.51819 (16)	0.0256 (7)	
H27	0.6836	0.4324	0.4878	0.031*	
C28	0.6797 (3)	0.4585 (2)	0.57628 (16)	0.0268 (7)	
H28	0.7068	0.4047	0.5853	0.032*	
C29	0.5096 (3)	0.8831 (2)	0.6994 (2)	0.0358 (9)	
H29A	0.4359	0.8875	0.7078	0.054*	
H29B	0.5225	0.9001	0.6593	0.054*	
H29C	0.5524	0.9226	0.7282	0.054*	
C30	0.6202 (3)	0.5746 (2)	0.43989 (17)	0.0294 (8)	
H30A	0.5693	0.6188	0.4363	0.044*	
H30B	0.5969	0.5207	0.4151	0.044*	
H30C	0.6879	0.5978	0.4268	0.044*	
C31	0.4418 (3)	0.4455 (2)	0.71593 (17)	0.0271 (8)	
H31	0.4352	0.5045	0.7315	0.032*	
C32	0.3516 (3)	0.3899 (3)	0.70505 (17)	0.0309 (8)	
H32	0.2851	0.4099	0.7139	0.037*	
C33	0.3620 (3)	0.3039 (3)	0.68068 (18)	0.0326 (8)	
C34	0.4617 (3)	0.2751 (2)	0.67357 (18)	0.0311 (8)	
H34	0.4704	0.2157	0.6596	0.037*	
C35	0.5474 (3)	0.3335 (3)	0.68687 (18)	0.0311 (8)	
C36	0.6546 (3)	0.3095 (3)	0.68455 (17)	0.0316 (8)	
C37	0.6810 (3)	0.2242 (3)	0.67203 (17)	0.0322 (8)	
H37	0.6279	0.1781	0.6635	0.039*	
C38	0.7843 (3)	0.2053 (3)	0.67183 (16)	0.0293 (8)	
C39	0.8597 (3)	0.2777 (3)	0.68264 (16)	0.0311 (8)	
H39	0.9317	0.2685	0.6808	0.037*	
C40	0.8283 (3)	0.3611 (2)	0.69583 (15)	0.0265 (7)	
H40	0.8793	0.4089	0.7041	0.032*	
C41	0.2669 (3)	0.2431 (3)	0.66303 (19)	0.0337 (9)	
H41A	0.2057	0.2669	0.6814	0.051*	
H41B	0.2763	0.1846	0.6764	0.051*	
H41C	0.2568	0.2383	0.6198	0.051*	
C42	0.8150 (3)	0.1134 (3)	0.66027 (19)	0.0362 (9)	
H42A	0.8135	0.0842	0.6972	0.054*	
H42B	0.8855	0.1145	0.6451	0.054*	
H42C	0.7663	0.0809	0.6309	0.054*	
C43	−0.0646 (3)	0.9013 (2)	0.71790 (16)	0.0257 (7)	
H43	−0.0415	0.9245	0.6825	0.031*	
C44	−0.1584 (3)	0.8493 (3)	0.71630 (17)	0.0306 (8)	
H44	−0.1968	0.8354	0.6799	0.037*	
C45	−0.1944 (3)	0.8186 (3)	0.76692 (17)	0.0285 (8)	
H45	−0.2589	0.7842	0.7661	0.034*	
C46	−0.1352 (3)	0.8378 (3)	0.82211 (17)	0.0312 (8)	
C47	−0.0412 (3)	0.8879 (3)	0.81962 (17)	0.0303 (8)	
C48	0.0190 (3)	0.9115 (2)	0.87106 (16)	0.0277 (8)	
C49	−0.0148 (3)	0.8850 (2)	0.92500 (17)	0.0278 (8)	
C50	0.0524 (3)	0.9125 (2)	0.97604 (16)	0.0266 (8)	
H50	0.0339	0.8954	1.0140	0.032*	
C51	0.1444 (3)	0.9642 (2)	0.96987 (16)	0.0238 (7)	
H51	0.1888	0.9827	1.0034	0.029*	
C52	0.1703 (3)	0.9883 (3)	0.91402 (17)	0.0307 (8)	
H52	0.2325	1.0245	0.9103	0.037*	
C53	−0.1689 (3)	0.8113 (3)	0.87605 (17)	0.0302 (8)	
C54	−0.1087 (3)	0.8349 (2)	0.92749 (17)	0.0280 (8)	
C55	−0.2980 (3)	0.7442 (2)	0.92976 (16)	0.0273 (8)	
C56	−0.4072 (3)	0.7008 (3)	0.93102 (17)	0.0287 (8)	
H56A	−0.4159	0.6522	0.8991	0.034*	
H56B	−0.4576	0.7445	0.9227	0.034*	
C57	−0.4323 (3)	0.6639 (2)	0.99009 (17)	0.0301 (8)	
H57A	−0.5087	0.6534	0.9927	0.036*	
H57B	−0.4012	0.6066	0.9921	0.036*	
C58	−0.3912 (4)	0.7260 (3)	1.04113 (18)	0.0395 (10)	
H58A	−0.4121	0.7018	1.0786	0.047*	
H58B	−0.4225	0.7832	1.0391	0.047*	
C59	−0.2727 (3)	0.7412 (3)	1.04133 (18)	0.0380 (9)	
H59A	−0.2496	0.7887	1.0723	0.046*	
H59B	−0.2411	0.6867	1.0512	0.046*	
C60	−0.2355 (3)	0.7662 (2)	0.98227 (16)	0.0235 (7)	
C61	−0.0141 (3)	1.1334 (3)	0.80113 (17)	0.0289 (8)	
H61	−0.0670	1.0865	0.7977	0.035*	
C62	−0.0428 (3)	1.2198 (2)	0.81099 (15)	0.0250 (7)	
H62	−0.1142	1.2309	0.8139	0.030*	
C63	0.0333 (3)	1.2896 (2)	0.81656 (16)	0.0265 (8)	
C64	0.1388 (3)	1.2683 (2)	0.81322 (17)	0.0281 (8)	
H64	0.1934	1.3139	0.8175	0.034*	
C65	0.1617 (3)	1.1814 (2)	0.80383 (15)	0.0234 (7)	
C66	0.2676 (3)	1.1526 (3)	0.80295 (17)	0.0299 (8)	
C67	0.3566 (3)	1.2104 (3)	0.81180 (18)	0.0338 (9)	
H67	0.3499	1.2722	0.8163	0.041*	
C68	0.4548 (3)	1.1787 (3)	0.81404 (18)	0.0309 (8)	
C69	0.4597 (3)	1.0869 (2)	0.80538 (16)	0.0262 (7)	
H69	0.5252	1.0619	0.8055	0.031*	
C70	0.3708 (3)	1.0343 (2)	0.79682 (18)	0.0308 (8)	
H70	0.3759	0.9724	0.7917	0.037*	
C71	0.0082 (3)	1.3844 (3)	0.82392 (19)	0.0376 (9)	
H71A	0.0217	1.4119	0.7871	0.056*	
H71B	0.0518	1.4155	0.8562	0.056*	
H71C	−0.0653	1.3875	0.8332	0.056*	
C72	0.5523 (3)	1.2393 (3)	0.82547 (19)	0.0350 (9)	
H72A	0.5378	1.2992	0.8169	0.052*	
H72B	0.6072	1.2184	0.7999	0.052*	
H72C	0.5752	1.2395	0.8671	0.052*	
C73	0.1152 (3)	1.0610 (2)	0.65762 (17)	0.0291 (8)	
H73	0.0956	1.1136	0.6784	0.035*	
C74	0.1111 (3)	1.0536 (3)	0.59582 (17)	0.0326 (8)	
H74	0.0895	1.1013	0.5752	0.039*	
C75	0.1379 (3)	0.9783 (2)	0.56454 (16)	0.0260 (7)	
C76	0.1712 (3)	0.9119 (3)	0.59719 (16)	0.0311 (8)	
H76	0.1912	0.8587	0.5773	0.037*	
C77	0.1755 (3)	0.9223 (2)	0.65848 (16)	0.0284 (8)	
C78	0.2071 (3)	0.8543 (2)	0.69516 (17)	0.0276 (8)	
C79	0.2403 (3)	0.7738 (2)	0.67269 (18)	0.0313 (8)	
H79	0.2417	0.7606	0.6310	0.038*	
C80	0.2715 (3)	0.7123 (2)	0.71034 (16)	0.0259 (7)	
C81	0.2704 (3)	0.7356 (3)	0.77169 (18)	0.0325 (8)	
H81	0.2931	0.6966	0.7991	0.039*	
C82	0.2359 (3)	0.8158 (2)	0.79130 (17)	0.0265 (7)	
H82	0.2353	0.8309	0.8328	0.032*	
C83	0.1309 (3)	0.9659 (3)	0.49803 (17)	0.0314 (8)	
H83A	0.0729	0.9227	0.4854	0.047*	
H83B	0.1960	0.9445	0.4832	0.047*	
H83C	0.1192	1.0223	0.4822	0.047*	
C84	0.3023 (3)	0.6245 (2)	0.68666 (18)	0.0313 (8)	
H84A	0.2541	0.6008	0.6541	0.047*	
H84B	0.3000	0.5843	0.7182	0.047*	
H84C	0.3731	0.6305	0.6721	0.047*	
C85	0.3876 (3)	0.0817 (3)	0.51856 (19)	0.0360 (9)	
H85A	0.3474	0.1339	0.5185	0.054*	
H85B	0.3472	0.0310	0.4978	0.054*	
H85C	0.4530	0.0927	0.4984	0.054*	
C86	0.4104 (3)	0.0630 (3)	0.57991 (19)	0.0375 (10)	
C87	0.0649 (3)	0.4508 (3)	0.56564 (19)	0.0371 (9)	
H87A	0.0838	0.5135	0.5621	0.056*	
H87B	−0.0067	0.4360	0.5506	0.056*	
H87C	0.1122	0.4151	0.5425	0.056*	
C88	0.0730 (3)	0.4331 (3)	0.62634 (19)	0.0372 (9)	
Cl1	0.72850 (8)	0.04379 (7)	0.86549 (4)	0.0386 (2)	
Cl2	0.64092 (8)	0.22425 (7)	0.50666 (5)	0.0422 (2)	
Cl3	0.85406 (8)	0.76754 (7)	0.54134 (5)	0.0407 (2)	
Cl4	0.32897 (9)	0.50337 (7)	0.86779 (5)	0.0400 (2)	
N1	0.6955 (2)	0.5037 (2)	0.80052 (13)	0.0268 (6)	
N2	0.8133 (2)	0.57237 (19)	0.72028 (14)	0.0253 (6)	
N3	0.9495 (2)	0.6048 (2)	0.95465 (14)	0.0275 (6)	
N4	1.0769 (2)	0.67485 (19)	0.86966 (13)	0.0257 (6)	
N5	0.6017 (2)	0.6185 (2)	0.71154 (14)	0.0263 (6)	
N6	0.6546 (2)	0.5167 (2)	0.62144 (13)	0.0279 (7)	
N7	0.5364 (2)	0.4201 (2)	0.70566 (15)	0.0304 (7)	
N8	0.7274 (2)	0.3765 (2)	0.69733 (14)	0.0281 (7)	
N9	−0.0061 (2)	0.9196 (2)	0.76794 (14)	0.0287 (7)	
N10	0.1102 (3)	0.9622 (2)	0.86422 (14)	0.0301 (7)	
N11	−0.2641 (3)	0.7642 (2)	0.87774 (14)	0.0288 (7)	
N12	−0.1434 (3)	0.8115 (2)	0.98090 (15)	0.0329 (7)	
N13	0.0856 (2)	1.1145 (2)	0.79637 (14)	0.0286 (7)	
N14	0.2744 (2)	1.0653 (2)	0.79511 (14)	0.0277 (7)	
N15	0.2027 (3)	0.8746 (2)	0.75447 (14)	0.0296 (7)	
N16	0.1465 (2)	0.9952 (2)	0.68870 (14)	0.0283 (7)	
N17	0.4256 (3)	0.0544 (2)	0.62973 (17)	0.0384 (8)	
N18	0.0842 (3)	0.4204 (2)	0.67795 (16)	0.0352 (8)	
O1W	0.5927 (6)	0.0437 (6)	0.0153 (4)	0.049 (3)	0.398 (9)
H1X	0.5943	0.0625	0.0517	0.059*	0.398 (9)
H1Y	0.5708	−0.0104	0.0115	0.059*	0.398 (9)
O2W	0.8861 (7)	0.3301 (6)	0.4446 (4)	0.036 (3)	0.312 (8)
H2X	0.9508	0.3444	0.4429	0.043*	0.312 (8)
H2Y	0.8763	0.2927	0.4700	0.043*	0.312 (8)
O3W	0.5260 (8)	0.1134 (6)	0.1236 (4)	0.038 (3)	0.290 (8)
H3X	0.4614	0.1158	0.1295	0.045*	0.290 (8)
H3Y	0.5426	0.1433	0.0948	0.045*	0.290 (8)
O11	0.6741 (2)	0.09701 (18)	0.90694 (13)	0.0369 (6)	
O12	0.6665 (2)	−0.0385 (2)	0.85157 (13)	0.0430 (7)	
O13	0.8304 (2)	0.0304 (2)	0.88772 (14)	0.0416 (7)	
O14	0.7385 (2)	0.0897 (2)	0.81302 (13)	0.0416 (7)	
O21	0.6442 (2)	0.13415 (19)	0.51944 (13)	0.0405 (7)	
O22	0.6258 (2)	0.2394 (2)	0.44767 (13)	0.0425 (7)	
O23	0.7409 (2)	0.2653 (2)	0.52630 (14)	0.0445 (7)	
O24	0.5645 (2)	0.2643 (2)	0.54161 (14)	0.0424 (7)	
O31	0.8783 (2)	0.6801 (2)	0.55237 (14)	0.0440 (7)	
O32	0.8260 (3)	0.7712 (2)	0.48109 (14)	0.0446 (7)	
O33	0.9370 (3)	0.8284 (2)	0.56377 (15)	0.0521 (8)	
O34	0.7682 (3)	0.7895 (2)	0.57360 (15)	0.0464 (8)	
O41	0.3766 (2)	0.4243 (2)	0.85520 (13)	0.0418 (7)	
O42	0.2436 (3)	0.4996 (2)	0.82055 (15)	0.0511 (8)	
O43	0.3916 (2)	0.57706 (19)	0.85471 (14)	0.0403 (7)	
O44	0.2729 (3)	0.5058 (2)	0.91728 (14)	0.0491 (8)	
Ru1	0.67096 (2)	0.500866 (18)	0.710312 (12)	0.02118 (8)	
Ru2	0.13664 (2)	0.989837 (19)	0.778743 (13)	0.02467 (9)	

### Atomic displacement parameters (Å^2^)

**Table:**

	*U*^11^	*U*^22^	*U*^33^	*U*^12^	*U*^13^	*U*^23^
C1	0.0245 (18)	0.041 (2)	0.031 (2)	0.0053 (16)	0.0004 (15)	0.0075 (17)
C2	0.0294 (19)	0.035 (2)	0.029 (2)	0.0033 (16)	0.0017 (15)	0.0021 (16)
C3	0.0286 (19)	0.0300 (19)	0.0276 (19)	0.0083 (15)	−0.0025 (15)	0.0036 (15)
C4	0.0239 (18)	0.0317 (19)	0.031 (2)	0.0049 (15)	−0.0044 (15)	0.0000 (15)
C5	0.0239 (18)	0.0312 (19)	0.031 (2)	0.0015 (15)	−0.0041 (15)	0.0059 (15)
C6	0.0194 (17)	0.036 (2)	0.0304 (19)	0.0051 (15)	−0.0029 (14)	0.0021 (16)
C7	0.0196 (16)	0.0239 (17)	0.0321 (19)	0.0064 (13)	−0.0012 (14)	0.0055 (14)
C8	0.0251 (18)	0.034 (2)	0.033 (2)	0.0014 (15)	0.0023 (15)	0.0040 (16)
C9	0.0195 (17)	0.041 (2)	0.032 (2)	−0.0001 (15)	−0.0019 (15)	0.0049 (17)
C10	0.0261 (19)	0.0296 (19)	0.035 (2)	−0.0029 (15)	0.0031 (15)	0.0061 (16)
C11	0.0288 (19)	0.0292 (19)	0.0291 (19)	0.0016 (15)	−0.0030 (15)	−0.0005 (15)
C12	0.0228 (17)	0.0305 (19)	0.0265 (18)	0.0089 (14)	0.0004 (14)	−0.0030 (15)
C13	0.0213 (16)	0.0183 (16)	0.0240 (17)	0.0094 (13)	−0.0019 (13)	0.0037 (13)
C14	0.0297 (19)	0.033 (2)	0.0281 (19)	0.0077 (16)	−0.0039 (15)	−0.0015 (15)
C15	0.035 (2)	0.0296 (19)	0.030 (2)	0.0091 (16)	−0.0071 (16)	−0.0102 (16)
C16	0.0295 (19)	0.037 (2)	0.0280 (19)	0.0076 (16)	−0.0072 (15)	−0.0014 (16)
C17	0.0184 (17)	0.0257 (17)	0.035 (2)	0.0061 (14)	−0.0007 (14)	0.0031 (15)
C18	0.0198 (17)	0.0192 (16)	0.0327 (19)	0.0062 (13)	−0.0015 (14)	0.0050 (14)
C19	0.0256 (18)	0.0238 (17)	0.0266 (18)	−0.0019 (14)	−0.0030 (14)	−0.0019 (14)
C20	0.0273 (18)	0.0241 (17)	0.0268 (18)	−0.0006 (14)	−0.0004 (14)	−0.0021 (14)
C21	0.0203 (17)	0.0241 (17)	0.0313 (19)	−0.0014 (14)	0.0011 (14)	0.0012 (14)
C22	0.0273 (18)	0.032 (2)	0.0261 (18)	0.0049 (15)	0.0013 (14)	0.0036 (15)
C23	0.034 (2)	0.0270 (18)	0.032 (2)	0.0005 (15)	0.0003 (16)	0.0034 (15)
C24	0.039 (2)	0.031 (2)	0.030 (2)	0.0058 (17)	0.0010 (16)	0.0057 (16)
C25	0.0266 (19)	0.033 (2)	0.032 (2)	0.0050 (15)	0.0020 (15)	0.0062 (16)
C26	0.0194 (16)	0.0219 (16)	0.0278 (18)	0.0007 (13)	0.0011 (13)	0.0099 (14)
C27	0.0302 (18)	0.0262 (18)	0.0219 (17)	0.0054 (14)	−0.0004 (14)	0.0082 (14)
C28	0.0259 (18)	0.0287 (18)	0.0263 (18)	0.0052 (15)	−0.0027 (14)	0.0036 (15)
C29	0.035 (2)	0.0235 (19)	0.048 (2)	0.0019 (16)	0.0028 (18)	0.0022 (17)
C30	0.034 (2)	0.0266 (18)	0.030 (2)	0.0064 (15)	−0.0004 (15)	0.0107 (15)
C31	0.0242 (18)	0.0249 (18)	0.033 (2)	0.0007 (14)	−0.0003 (15)	0.0120 (15)
C32	0.0287 (19)	0.032 (2)	0.032 (2)	−0.0041 (15)	−0.0032 (15)	0.0090 (16)
C33	0.030 (2)	0.034 (2)	0.033 (2)	−0.0066 (16)	−0.0036 (16)	0.0052 (16)
C34	0.034 (2)	0.0222 (18)	0.036 (2)	−0.0063 (15)	0.0002 (16)	0.0034 (15)
C35	0.030 (2)	0.0290 (19)	0.033 (2)	−0.0006 (15)	−0.0028 (15)	0.0022 (16)
C36	0.034 (2)	0.0293 (19)	0.032 (2)	0.0039 (16)	0.0004 (16)	0.0024 (16)
C37	0.039 (2)	0.0279 (19)	0.030 (2)	0.0084 (16)	−0.0075 (16)	0.0028 (15)
C38	0.038 (2)	0.0309 (19)	0.0193 (17)	0.0127 (16)	−0.0056 (15)	0.0000 (14)
C39	0.038 (2)	0.033 (2)	0.0234 (18)	0.0122 (17)	−0.0014 (15)	0.0014 (15)
C40	0.0307 (19)	0.0311 (19)	0.0199 (17)	0.0108 (15)	0.0014 (14)	0.0084 (14)
C41	0.030 (2)	0.032 (2)	0.037 (2)	−0.0015 (16)	0.0014 (16)	−0.0009 (17)
C42	0.033 (2)	0.032 (2)	0.041 (2)	0.0057 (17)	−0.0011 (17)	−0.0109 (17)
C43	0.0305 (19)	0.0227 (17)	0.0234 (18)	0.0027 (14)	−0.0013 (14)	−0.0004 (14)
C44	0.032 (2)	0.033 (2)	0.0266 (19)	0.0004 (16)	−0.0053 (15)	0.0085 (16)
C45	0.0219 (17)	0.0326 (19)	0.031 (2)	0.0033 (15)	0.0002 (14)	0.0030 (15)
C46	0.033 (2)	0.0296 (19)	0.030 (2)	−0.0023 (16)	−0.0002 (16)	−0.0031 (16)
C47	0.031 (2)	0.0310 (19)	0.0289 (19)	0.0035 (15)	−0.0017 (15)	0.0006 (15)
C48	0.0213 (17)	0.0330 (19)	0.0296 (19)	0.0084 (15)	−0.0020 (14)	0.0034 (15)
C49	0.0248 (18)	0.0288 (18)	0.0291 (19)	0.0032 (15)	−0.0026 (14)	−0.0012 (15)
C50	0.0203 (17)	0.037 (2)	0.0234 (18)	0.0119 (15)	−0.0039 (13)	0.0003 (15)
C51	0.0220 (17)	0.0254 (17)	0.0243 (17)	0.0094 (14)	−0.0079 (13)	−0.0001 (14)
C52	0.0268 (19)	0.036 (2)	0.0294 (19)	0.0054 (16)	−0.0025 (15)	0.0009 (16)
C53	0.0252 (19)	0.032 (2)	0.033 (2)	0.0032 (15)	−0.0038 (15)	0.0030 (16)
C54	0.0225 (18)	0.0316 (19)	0.0303 (19)	0.0063 (15)	−0.0003 (14)	0.0011 (15)
C55	0.0233 (18)	0.033 (2)	0.0252 (18)	0.0047 (15)	−0.0005 (14)	0.0006 (15)
C56	0.0253 (18)	0.0323 (19)	0.0292 (19)	0.0085 (15)	−0.0014 (14)	0.0020 (15)
C57	0.0282 (19)	0.0273 (19)	0.035 (2)	0.0001 (15)	−0.0027 (15)	0.0072 (16)
C58	0.049 (3)	0.042 (2)	0.026 (2)	−0.0082 (19)	0.0073 (18)	0.0025 (17)
C59	0.044 (2)	0.039 (2)	0.030 (2)	−0.0083 (19)	−0.0028 (18)	0.0058 (17)
C60	0.0234 (17)	0.0232 (17)	0.0243 (17)	0.0078 (14)	−0.0036 (13)	0.0007 (13)
C61	0.0265 (19)	0.0301 (19)	0.0300 (19)	0.0060 (15)	−0.0020 (15)	0.0001 (15)
C62	0.0261 (18)	0.0309 (18)	0.0186 (17)	0.0124 (15)	−0.0056 (13)	−0.0007 (14)
C63	0.0336 (19)	0.0252 (18)	0.0211 (17)	0.0136 (15)	−0.0047 (14)	−0.0026 (14)
C64	0.0303 (19)	0.0231 (17)	0.0317 (19)	0.0073 (15)	−0.0046 (15)	0.0043 (15)
C65	0.0240 (17)	0.0230 (17)	0.0240 (17)	0.0040 (14)	−0.0013 (13)	0.0042 (14)
C66	0.0241 (18)	0.0307 (19)	0.035 (2)	0.0038 (15)	−0.0028 (15)	−0.0002 (16)
C67	0.027 (2)	0.033 (2)	0.040 (2)	0.0017 (16)	−0.0043 (16)	−0.0026 (17)
C68	0.0234 (18)	0.0316 (19)	0.037 (2)	−0.0029 (15)	−0.0027 (15)	0.0043 (16)
C69	0.0218 (17)	0.0271 (18)	0.0287 (19)	−0.0006 (14)	0.0020 (14)	−0.0014 (14)
C70	0.0267 (19)	0.0254 (18)	0.039 (2)	0.0021 (15)	−0.0055 (16)	−0.0050 (16)
C71	0.044 (2)	0.028 (2)	0.040 (2)	0.0169 (18)	−0.0083 (18)	−0.0064 (17)
C72	0.031 (2)	0.032 (2)	0.040 (2)	−0.0059 (16)	−0.0076 (17)	0.0034 (17)
C73	0.034 (2)	0.0242 (18)	0.0287 (19)	0.0022 (15)	−0.0023 (15)	0.0027 (15)
C74	0.037 (2)	0.032 (2)	0.029 (2)	−0.0010 (16)	0.0001 (16)	0.0023 (16)
C75	0.0200 (17)	0.0348 (19)	0.0220 (17)	−0.0041 (14)	−0.0014 (13)	0.0011 (15)
C76	0.034 (2)	0.035 (2)	0.0230 (18)	0.0040 (16)	0.0019 (15)	−0.0017 (15)
C77	0.036 (2)	0.0232 (17)	0.0261 (19)	0.0038 (15)	0.0038 (15)	0.0018 (14)
C78	0.0289 (19)	0.0221 (17)	0.0313 (19)	−0.0008 (14)	0.0002 (15)	0.0011 (15)
C79	0.033 (2)	0.0247 (18)	0.036 (2)	0.0050 (15)	0.0002 (16)	−0.0008 (16)
C80	0.0230 (17)	0.0235 (17)	0.0321 (19)	0.0020 (14)	0.0085 (14)	0.0040 (15)
C81	0.036 (2)	0.0269 (19)	0.035 (2)	0.0041 (16)	0.0049 (16)	0.0037 (16)
C82	0.0184 (17)	0.0308 (19)	0.0306 (19)	0.0024 (14)	−0.0004 (14)	0.0042 (15)
C83	0.032 (2)	0.035 (2)	0.028 (2)	0.0117 (16)	−0.0021 (15)	0.0023 (16)
C84	0.034 (2)	0.0277 (19)	0.033 (2)	0.0106 (16)	0.0033 (16)	−0.0008 (16)
C85	0.030 (2)	0.036 (2)	0.039 (2)	−0.0011 (17)	−0.0067 (17)	−0.0037 (17)
C86	0.035 (2)	0.034 (2)	0.041 (2)	0.0089 (17)	−0.0144 (18)	−0.0122 (18)
C87	0.043 (2)	0.032 (2)	0.042 (2)	0.0171 (18)	0.0164 (18)	0.0187 (18)
C88	0.038 (2)	0.036 (2)	0.041 (2)	0.0145 (18)	−0.0039 (18)	0.0093 (18)
Cl1	0.0431 (6)	0.0385 (5)	0.0344 (5)	0.0143 (4)	−0.0025 (4)	−0.0052 (4)
Cl2	0.0374 (5)	0.0424 (6)	0.0474 (6)	0.0114 (4)	0.0104 (4)	−0.0018 (5)
Cl3	0.0452 (6)	0.0451 (6)	0.0321 (5)	−0.0044 (5)	−0.0013 (4)	0.0117 (4)
Cl4	0.0446 (6)	0.0408 (5)	0.0345 (5)	−0.0008 (4)	0.0143 (4)	0.0004 (4)
N1	0.0208 (15)	0.0376 (17)	0.0223 (15)	0.0076 (13)	−0.0015 (11)	0.0007 (13)
N2	0.0240 (15)	0.0217 (14)	0.0305 (16)	0.0022 (12)	−0.0012 (12)	0.0052 (12)
N3	0.0220 (15)	0.0276 (16)	0.0327 (17)	0.0007 (12)	−0.0027 (12)	0.0031 (13)
N4	0.0324 (16)	0.0201 (14)	0.0248 (15)	0.0034 (12)	0.0011 (12)	0.0020 (12)
N5	0.0216 (15)	0.0257 (15)	0.0316 (16)	0.0013 (12)	0.0028 (12)	0.0020 (13)
N6	0.0242 (15)	0.0346 (17)	0.0245 (16)	0.0041 (13)	−0.0045 (12)	0.0003 (13)
N7	0.0263 (16)	0.0286 (16)	0.0353 (18)	−0.0023 (13)	−0.0054 (13)	0.0028 (13)
N8	0.0303 (16)	0.0234 (15)	0.0313 (17)	0.0053 (12)	0.0009 (13)	0.0037 (13)
N9	0.0223 (15)	0.0332 (17)	0.0299 (16)	0.0031 (13)	−0.0004 (12)	−0.0025 (13)
N10	0.0335 (17)	0.0332 (17)	0.0242 (16)	0.0018 (13)	−0.0028 (13)	0.0069 (13)
N11	0.0345 (17)	0.0224 (15)	0.0293 (17)	−0.0006 (13)	−0.0034 (13)	0.0039 (13)
N12	0.0295 (17)	0.0352 (18)	0.0340 (18)	0.0016 (14)	−0.0016 (13)	0.0046 (14)
N13	0.0281 (16)	0.0242 (15)	0.0337 (17)	0.0066 (12)	−0.0015 (13)	0.0005 (13)
N14	0.0214 (15)	0.0308 (16)	0.0298 (16)	0.0023 (12)	−0.0036 (12)	−0.0035 (13)
N15	0.0328 (17)	0.0234 (15)	0.0326 (17)	0.0034 (13)	0.0000 (13)	0.0015 (13)
N16	0.0303 (17)	0.0275 (16)	0.0265 (16)	0.0008 (13)	−0.0025 (12)	0.0004 (13)
N17	0.0371 (19)	0.0314 (18)	0.045 (2)	0.0033 (15)	−0.0169 (16)	−0.0018 (15)
N18	0.0343 (18)	0.0368 (18)	0.038 (2)	0.0128 (14)	0.0144 (14)	0.0123 (15)
O1W	0.047 (5)	0.058 (5)	0.042 (5)	0.014 (4)	−0.016 (4)	0.006 (4)
O2W	0.030 (5)	0.037 (5)	0.038 (5)	−0.005 (4)	−0.001 (4)	−0.002 (4)
O3W	0.046 (6)	0.036 (6)	0.034 (6)	0.010 (4)	−0.001 (4)	0.019 (4)
O11	0.0340 (15)	0.0323 (15)	0.0448 (17)	0.0086 (12)	0.0080 (12)	−0.0020 (12)
O12	0.0414 (17)	0.0441 (17)	0.0392 (17)	0.0020 (13)	−0.0056 (13)	−0.0181 (13)
O13	0.0350 (16)	0.0416 (16)	0.0479 (18)	0.0051 (13)	−0.0038 (13)	0.0018 (14)
O14	0.0343 (16)	0.0519 (18)	0.0387 (16)	0.0072 (13)	0.0042 (12)	0.0008 (14)
O21	0.0390 (16)	0.0369 (16)	0.0491 (18)	0.0159 (13)	0.0295 (14)	0.0053 (13)
O22	0.0414 (17)	0.0452 (17)	0.0447 (17)	0.0326 (14)	0.0069 (13)	−0.0003 (14)
O23	0.0423 (17)	0.0433 (17)	0.0472 (18)	0.0005 (14)	0.0075 (14)	0.0007 (14)
O24	0.0418 (17)	0.0401 (16)	0.0454 (18)	0.0120 (13)	0.0093 (13)	−0.0064 (13)
O31	0.0488 (18)	0.0465 (18)	0.0410 (17)	0.0154 (14)	0.0112 (14)	0.0148 (14)
O32	0.0486 (18)	0.0477 (18)	0.0401 (17)	0.0127 (14)	−0.0006 (14)	0.0118 (14)
O33	0.0455 (19)	0.057 (2)	0.053 (2)	−0.0137 (15)	−0.0121 (15)	0.0161 (16)
O34	0.0454 (18)	0.0415 (17)	0.0494 (19)	0.0047 (14)	−0.0013 (14)	−0.0134 (14)
O41	0.0457 (17)	0.0391 (16)	0.0410 (17)	0.0030 (13)	0.0177 (14)	0.0009 (13)
O42	0.057 (2)	0.0449 (18)	0.0481 (19)	0.0045 (15)	−0.0174 (16)	−0.0111 (15)
O43	0.0429 (17)	0.0371 (16)	0.0437 (17)	0.0056 (13)	0.0083 (13)	0.0153 (13)
O44	0.054 (2)	0.053 (2)	0.0438 (18)	0.0150 (16)	0.0230 (15)	0.0112 (15)
Ru1	0.01880 (14)	0.02331 (15)	0.02158 (15)	0.00198 (10)	−0.00196 (10)	0.00327 (10)
Ru2	0.02332 (15)	0.02394 (15)	0.02642 (16)	0.00243 (11)	−0.00156 (11)	0.00031 (11)

### Geometric parameters (Å, °)

**Table:**

C1—N1	1.352 (5)	C53—C54	1.390 (5)
C1—C2	1.401 (5)	C54—N12	1.368 (5)
C1—H1	0.9500	C55—N11	1.320 (5)
C2—C3	1.383 (5)	C55—C60	1.421 (5)
C2—H2	0.9500	C55—C56	1.514 (5)
C3—C4	1.451 (5)	C56—C57	1.528 (5)
C3—H3	0.9500	C56—H56A	0.9900
C4—C5	1.390 (5)	C56—H56B	0.9900
C4—C11	1.390 (5)	C57—C58	1.493 (6)
C5—N1	1.378 (5)	C57—H57A	0.9900
C5—C6	1.390 (5)	C57—H57B	0.9900
C6—N2	1.384 (5)	C58—C59	1.529 (6)
C6—C7	1.390 (5)	C58—H58A	0.9900
C7—C12	1.390 (5)	C58—H58B	0.9900
C7—C8	1.461 (5)	C59—C60	1.503 (5)
C8—C9	1.383 (5)	C59—H59A	0.9900
C8—H8	0.9500	C59—H59B	0.9900
C9—C10	1.363 (5)	C60—N12	1.333 (5)
C9—H9	0.9500	C61—N13	1.342 (5)
C10—N2	1.335 (5)	C61—C62	1.395 (5)
C10—H10	0.9500	C61—H61	0.9500
C11—N3	1.358 (5)	C62—C63	1.392 (5)
C11—C12	1.390 (5)	C62—H62	0.9500
C12—N4	1.369 (5)	C63—C64	1.422 (5)
C13—N3	1.334 (5)	C63—C71	1.501 (5)
C13—C18	1.417 (5)	C64—C65	1.379 (5)
C13—C14	1.510 (5)	C64—H64	0.9500
C14—C15	1.511 (5)	C65—N13	1.362 (5)
C14—H14A	0.9900	C65—C66	1.463 (5)
C14—H14B	0.9900	C66—N14	1.339 (5)
C15—C16	1.532 (5)	C66—C67	1.396 (5)
C15—H15A	0.9900	C67—C68	1.388 (5)
C15—H15B	0.9900	C67—H67	0.9500
C16—C17	1.522 (5)	C68—C69	1.403 (5)
C16—H16A	0.9900	C68—C72	1.510 (5)
C16—H16B	0.9900	C69—C70	1.351 (5)
C17—C18	1.500 (5)	C69—H69	0.9500
C17—H17A	0.9900	C70—N14	1.361 (5)
C17—H17B	0.9900	C70—H70	0.9500
C18—N4	1.332 (5)	C71—H71A	0.9800
C19—N5	1.361 (5)	C71—H71B	0.9800
C19—C20	1.364 (5)	C71—H71C	0.9800
C19—H19	0.9500	C72—H72A	0.9800
C20—C21	1.407 (5)	C72—H72B	0.9800
C20—H20	0.9500	C72—H72C	0.9800
C21—C22	1.409 (5)	C73—N16	1.351 (5)
C21—C29	1.507 (5)	C73—C74	1.395 (5)
C22—C23	1.371 (5)	C73—H73	0.9500
C22—H22	0.9500	C74—C75	1.370 (6)
C23—N5	1.360 (5)	C74—H74	0.9500
C23—C24	1.481 (6)	C75—C76	1.388 (5)
C24—N6	1.339 (5)	C75—C83	1.503 (5)
C24—C25	1.383 (5)	C76—C77	1.384 (5)
C25—C26	1.386 (5)	C76—H76	0.9500
C25—H25	0.9500	C77—N16	1.338 (5)
C26—C27	1.388 (5)	C77—C78	1.455 (5)
C26—C30	1.497 (5)	C78—N15	1.358 (5)
C27—C28	1.369 (5)	C78—C79	1.389 (5)
C27—H27	0.9500	C79—C80	1.392 (5)
C28—N6	1.358 (5)	C79—H79	0.9500
C28—H28	0.9500	C80—C81	1.407 (5)
C29—H29A	0.9800	C80—C84	1.486 (5)
C29—H29B	0.9800	C81—C82	1.374 (5)
C29—H29C	0.9800	C81—H81	0.9500
C30—H30A	0.9800	C82—N15	1.359 (5)
C30—H30B	0.9800	C82—H82	0.9500
C30—H30C	0.9800	C83—H83A	0.9800
C31—N7	1.325 (5)	C83—H83B	0.9800
C31—C32	1.393 (5)	C83—H83C	0.9800
C31—H31	0.9500	C84—H84A	0.9800
C32—C33	1.398 (6)	C84—H84B	0.9800
C32—H32	0.9500	C84—H84C	0.9800
C33—C34	1.396 (6)	C85—C86	1.468 (6)
C33—C41	1.508 (5)	C85—H85A	0.9800
C34—C35	1.379 (5)	C85—H85B	0.9800
C34—H34	0.9500	C85—H85C	0.9800
C35—N7	1.374 (5)	C86—N17	1.161 (6)
C35—C36	1.455 (6)	C87—C88	1.426 (6)
C36—N8	1.345 (5)	C87—H87A	0.9800
C36—C37	1.377 (5)	C87—H87B	0.9800
C37—C38	1.382 (6)	C87—H87C	0.9800
C37—H37	0.9500	C88—N18	1.207 (5)
C38—C39	1.418 (6)	Cl1—O11	1.422 (3)
C38—C42	1.488 (5)	Cl1—O13	1.428 (3)
C39—C40	1.374 (5)	Cl1—O14	1.434 (3)
C39—H39	0.9500	Cl1—O12	1.447 (3)
C40—N8	1.338 (5)	Cl2—O22	1.386 (3)
C40—H40	0.9500	Cl2—O24	1.421 (3)
C41—H41A	0.9800	Cl2—O21	1.431 (3)
C41—H41B	0.9800	Cl2—O23	1.435 (3)
C41—H41C	0.9800	Cl3—O34	1.389 (3)
C42—H42A	0.9800	Cl3—O32	1.410 (3)
C42—H42B	0.9800	Cl3—O33	1.419 (3)
C42—H42C	0.9800	Cl3—O31	1.431 (3)
C43—N9	1.343 (5)	Cl4—O44	1.350 (3)
C43—C44	1.397 (5)	Cl4—O43	1.394 (3)
C43—H43	0.9500	Cl4—O41	1.404 (3)
C44—C45	1.355 (5)	Cl4—O42	1.508 (3)
C44—H44	0.9500	N1—Ru1	2.056 (3)
C45—C46	1.445 (5)	N2—Ru1	2.064 (3)
C45—H45	0.9500	N5—Ru1	2.058 (3)
C46—C47	1.390 (5)	N6—Ru1	2.055 (3)
C46—C53	1.390 (6)	N7—Ru1	2.050 (3)
C47—N9	1.379 (5)	N8—Ru1	2.081 (3)
C47—C48	1.390 (5)	N9—Ru2	2.059 (3)
C48—N10	1.381 (5)	N10—Ru2	2.048 (3)
C48—C49	1.390 (5)	N13—Ru2	2.068 (3)
C49—C54	1.390 (5)	N14—Ru2	2.054 (3)
C49—C50	1.441 (5)	N15—Ru2	2.048 (3)
C50—C51	1.394 (5)	N16—Ru2	2.056 (3)
C50—H50	0.9500	O1W—H1X	0.8499
C51—C52	1.390 (5)	O1W—H1Y	0.8500
C51—H51	0.9500	O2W—H2X	0.8500
C52—N10	1.366 (5)	O2W—H2Y	0.8501
C52—H52	0.9500	O3W—H3X	0.8501
C53—N11	1.381 (5)	O3W—H3Y	0.8498
			
N1—C1—C2	121.5 (4)	H58A—C58—H58B	107.9
N1—C1—H1	119.3	C60—C59—C58	111.6 (3)
C2—C1—H1	119.3	C60—C59—H59A	109.3
C3—C2—C1	119.8 (4)	C58—C59—H59A	109.3
C3—C2—H2	120.1	C60—C59—H59B	109.3
C1—C2—H2	120.1	C58—C59—H59B	109.3
C2—C3—C4	120.1 (3)	H59A—C59—H59B	108.0
C2—C3—H3	119.9	N12—C60—C55	121.1 (3)
C4—C3—H3	119.9	N12—C60—C59	118.0 (3)
C5—C4—C11	120.0 (4)	C55—C60—C59	120.9 (3)
C5—C4—C3	115.8 (3)	N13—C61—C62	122.3 (4)
C11—C4—C3	124.2 (3)	N13—C61—H61	118.9
N1—C5—C4	123.9 (3)	C62—C61—H61	118.9
N1—C5—C6	116.1 (3)	C63—C62—C61	119.8 (3)
C4—C5—C6	120.0 (3)	C63—C62—H62	120.1
N2—C6—C7	122.4 (3)	C61—C62—H62	120.1
N2—C6—C5	117.6 (3)	C62—C63—C64	117.3 (3)
C7—C6—C5	120.0 (3)	C62—C63—C71	122.9 (3)
C6—C7—C12	120.0 (3)	C64—C63—C71	119.8 (4)
C6—C7—C8	117.6 (3)	C65—C64—C63	119.8 (3)
C12—C7—C8	122.4 (3)	C65—C64—H64	120.1
C9—C8—C7	117.3 (3)	C63—C64—H64	120.1
C9—C8—H8	121.4	N13—C65—C64	121.7 (3)
C7—C8—H8	121.4	N13—C65—C66	114.3 (3)
C10—C9—C8	121.2 (4)	C64—C65—C66	123.9 (3)
C10—C9—H9	119.4	N14—C66—C67	121.1 (3)
C8—C9—H9	119.4	N14—C66—C65	115.4 (3)
N2—C10—C9	123.2 (4)	C67—C66—C65	123.4 (3)
N2—C10—H10	118.4	C68—C67—C66	120.7 (4)
C9—C10—H10	118.4	C68—C67—H67	119.6
N3—C11—C12	121.6 (3)	C66—C67—H67	119.6
N3—C11—C4	118.4 (4)	C67—C68—C69	116.9 (3)
C12—C11—C4	120.0 (3)	C67—C68—C72	122.0 (4)
N4—C12—C11	120.4 (3)	C69—C68—C72	121.1 (3)
N4—C12—C7	119.6 (3)	C70—C69—C68	119.6 (3)
C11—C12—C7	120.0 (3)	C70—C69—H69	120.2
N3—C13—C18	121.1 (3)	C68—C69—H69	120.2
N3—C13—C14	117.4 (3)	C69—C70—N14	123.6 (3)
C18—C13—C14	121.5 (3)	C69—C70—H70	118.2
C13—C14—C15	114.6 (3)	N14—C70—H70	118.2
C13—C14—H14A	108.6	C63—C71—H71A	109.5
C15—C14—H14A	108.6	C63—C71—H71B	109.5
C13—C14—H14B	108.6	H71A—C71—H71B	109.5
C15—C14—H14B	108.6	C63—C71—H71C	109.5
H14A—C14—H14B	107.6	H71A—C71—H71C	109.5
C14—C15—C16	110.9 (3)	H71B—C71—H71C	109.5
C14—C15—H15A	109.5	C68—C72—H72A	109.5
C16—C15—H15A	109.5	C68—C72—H72B	109.5
C14—C15—H15B	109.5	H72A—C72—H72B	109.5
C16—C15—H15B	109.5	C68—C72—H72C	109.5
H15A—C15—H15B	108.1	H72A—C72—H72C	109.5
C17—C16—C15	112.1 (3)	H72B—C72—H72C	109.5
C17—C16—H16A	109.2	N16—C73—C74	121.7 (3)
C15—C16—H16A	109.2	N16—C73—H73	119.2
C17—C16—H16B	109.2	C74—C73—H73	119.2
C15—C16—H16B	109.2	C75—C74—C73	120.6 (4)
H16A—C16—H16B	107.9	C75—C74—H74	119.7
C18—C17—C16	112.7 (3)	C73—C74—H74	119.7
C18—C17—H17A	109.0	C74—C75—C76	116.9 (3)
C16—C17—H17A	109.0	C74—C75—C83	122.6 (3)
C18—C17—H17B	109.0	C76—C75—C83	120.5 (3)
C16—C17—H17B	109.0	C77—C76—C75	120.7 (4)
H17A—C17—H17B	107.8	C77—C76—H76	119.7
N4—C18—C13	121.1 (3)	C75—C76—H76	119.7
N4—C18—C17	117.0 (3)	N16—C77—C76	122.0 (3)
C13—C18—C17	121.9 (3)	N16—C77—C78	114.7 (3)
N5—C19—C20	123.0 (3)	C76—C77—C78	123.2 (3)
N5—C19—H19	118.5	N15—C78—C79	121.0 (3)
C20—C19—H19	118.5	N15—C78—C77	115.1 (3)
C19—C20—C21	120.1 (3)	C79—C78—C77	123.9 (4)
C19—C20—H20	120.0	C78—C79—C80	121.0 (4)
C21—C20—H20	120.0	C78—C79—H79	119.5
C20—C21—C22	116.9 (3)	C80—C79—H79	119.5
C20—C21—C29	122.4 (3)	C79—C80—C81	117.5 (3)
C22—C21—C29	120.7 (3)	C79—C80—C84	121.3 (3)
C23—C22—C21	119.7 (3)	C81—C80—C84	121.2 (3)
C23—C22—H22	120.2	C82—C81—C80	118.9 (4)
C21—C22—H22	120.2	C82—C81—H81	120.6
N5—C23—C22	123.0 (4)	C80—C81—H81	120.6
N5—C23—C24	113.6 (3)	N15—C82—C81	123.5 (4)
C22—C23—C24	123.3 (4)	N15—C82—H82	118.3
N6—C24—C25	122.8 (4)	C81—C82—H82	118.3
N6—C24—C23	115.0 (3)	C75—C83—H83A	109.5
C25—C24—C23	122.2 (4)	C75—C83—H83B	109.5
C24—C25—C26	119.8 (4)	H83A—C83—H83B	109.5
C24—C25—H25	120.1	C75—C83—H83C	109.5
C26—C25—H25	120.1	H83A—C83—H83C	109.5
C25—C26—C27	116.9 (3)	H83B—C83—H83C	109.5
C25—C26—C30	121.8 (3)	C80—C84—H84A	109.5
C27—C26—C30	121.3 (3)	C80—C84—H84B	109.5
C28—C27—C26	120.9 (3)	H84A—C84—H84B	109.5
C28—C27—H27	119.6	C80—C84—H84C	109.5
C26—C27—H27	119.6	H84A—C84—H84C	109.5
N6—C28—C27	121.9 (3)	H84B—C84—H84C	109.5
N6—C28—H28	119.0	C86—C85—H85A	109.5
C27—C28—H28	119.0	C86—C85—H85B	109.5
C21—C29—H29A	109.5	H85A—C85—H85B	109.5
C21—C29—H29B	109.5	C86—C85—H85C	109.5
H29A—C29—H29B	109.5	H85A—C85—H85C	109.5
C21—C29—H29C	109.5	H85B—C85—H85C	109.5
H29A—C29—H29C	109.5	N17—C86—C85	174.8 (4)
H29B—C29—H29C	109.5	C88—C87—H87A	109.5
C26—C30—H30A	109.5	C88—C87—H87B	109.5
C26—C30—H30B	109.5	H87A—C87—H87B	109.5
H30A—C30—H30B	109.5	C88—C87—H87C	109.5
C26—C30—H30C	109.5	H87A—C87—H87C	109.5
H30A—C30—H30C	109.5	H87B—C87—H87C	109.5
H30B—C30—H30C	109.5	N18—C88—C87	177.0 (5)
N7—C31—C32	123.4 (4)	O11—Cl1—O13	111.40 (19)
N7—C31—H31	118.3	O11—Cl1—O14	107.43 (18)
C32—C31—H31	118.3	O13—Cl1—O14	108.39 (19)
C31—C32—C33	117.8 (4)	O11—Cl1—O12	108.10 (19)
C31—C32—H32	121.1	O13—Cl1—O12	112.05 (19)
C33—C32—H32	121.1	O14—Cl1—O12	109.35 (19)
C34—C33—C32	118.9 (3)	O22—Cl2—O24	110.19 (18)
C34—C33—C41	120.7 (4)	O22—Cl2—O21	116.57 (19)
C32—C33—C41	120.3 (4)	O24—Cl2—O21	109.14 (18)
C35—C34—C33	119.6 (4)	O22—Cl2—O23	107.7 (2)
C35—C34—H34	120.2	O24—Cl2—O23	107.91 (19)
C33—C34—H34	120.2	O21—Cl2—O23	104.90 (19)
N7—C35—C34	121.0 (4)	O34—Cl3—O32	106.8 (2)
N7—C35—C36	114.6 (3)	O34—Cl3—O33	105.6 (2)
C34—C35—C36	124.3 (4)	O32—Cl3—O33	115.5 (2)
N8—C36—C37	121.6 (4)	O34—Cl3—O31	109.0 (2)
N8—C36—C35	115.3 (3)	O32—Cl3—O31	110.4 (2)
C37—C36—C35	123.1 (4)	O33—Cl3—O31	109.3 (2)
C36—C37—C38	120.3 (4)	O44—Cl4—O43	120.4 (2)
C36—C37—H37	119.8	O44—Cl4—O41	114.4 (2)
C38—C37—H37	119.8	O43—Cl4—O41	112.45 (19)
C37—C38—C39	116.9 (3)	O44—Cl4—O42	100.9 (2)
C37—C38—C42	121.5 (4)	O43—Cl4—O42	101.8 (2)
C39—C38—C42	121.6 (3)	O41—Cl4—O42	103.4 (2)
C40—C39—C38	119.8 (4)	C1—N1—C5	118.9 (3)
C40—C39—H39	120.1	C1—N1—Ru1	127.1 (3)
C38—C39—H39	120.1	C5—N1—Ru1	114.0 (2)
N8—C40—C39	121.5 (4)	C10—N2—C6	118.3 (3)
N8—C40—H40	119.2	C10—N2—Ru1	128.9 (3)
C39—C40—H40	119.2	C6—N2—Ru1	112.7 (2)
C33—C41—H41A	109.5	C13—N3—C11	117.7 (3)
C33—C41—H41B	109.5	C18—N4—C12	118.0 (3)
H41A—C41—H41B	109.5	C23—N5—C19	117.2 (3)
C33—C41—H41C	109.5	C23—N5—Ru1	115.8 (2)
H41A—C41—H41C	109.5	C19—N5—Ru1	126.7 (2)
H41B—C41—H41C	109.5	C24—N6—C28	117.6 (3)
C38—C42—H42A	109.5	C24—N6—Ru1	116.3 (3)
C38—C42—H42B	109.5	C28—N6—Ru1	126.1 (2)
H42A—C42—H42B	109.5	C31—N7—C35	118.8 (3)
C38—C42—H42C	109.5	C31—N7—Ru1	125.8 (3)
H42A—C42—H42C	109.5	C35—N7—Ru1	115.2 (3)
H42B—C42—H42C	109.5	C40—N8—C36	119.6 (3)
N9—C43—C44	122.0 (3)	C40—N8—Ru1	124.8 (3)
N9—C43—H43	119.0	C36—N8—Ru1	115.3 (3)
C44—C43—H43	119.0	C43—N9—C47	118.8 (3)
C45—C44—C43	119.6 (3)	C43—N9—Ru2	128.0 (3)
C45—C44—H44	120.2	C47—N9—Ru2	113.2 (2)
C43—C44—H44	120.2	C52—N10—C48	117.4 (3)
C44—C45—C46	120.4 (4)	C52—N10—Ru2	128.2 (3)
C44—C45—H45	119.8	C48—N10—Ru2	114.3 (2)
C46—C45—H45	119.8	C55—N11—C53	118.3 (3)
C47—C46—C53	120.0 (4)	C60—N12—C54	118.6 (3)
C47—C46—C45	116.4 (4)	C61—N13—C65	119.0 (3)
C53—C46—C45	123.6 (4)	C61—N13—Ru2	125.6 (3)
N9—C47—C46	122.7 (3)	C65—N13—Ru2	115.5 (2)
N9—C47—C48	117.3 (3)	C66—N14—C70	118.0 (3)
C46—C47—C48	120.0 (4)	C66—N14—Ru2	116.2 (2)
N10—C48—C49	124.3 (3)	C70—N14—Ru2	125.7 (3)
N10—C48—C47	115.7 (3)	C78—N15—C82	118.1 (3)
C49—C48—C47	120.0 (3)	C78—N15—Ru2	115.1 (2)
C54—C49—C48	120.0 (3)	C82—N15—Ru2	126.6 (3)
C54—C49—C50	123.7 (4)	C77—N16—C73	118.1 (3)
C48—C49—C50	116.3 (3)	C77—N16—Ru2	115.9 (2)
C51—C50—C49	120.1 (3)	C73—N16—Ru2	125.6 (3)
C51—C50—H50	120.0	H1X—O1W—H1Y	109.5
C49—C50—H50	120.0	H2X—O2W—H2Y	109.5
C52—C51—C50	119.1 (3)	H3X—O3W—H3Y	109.5
C52—C51—H51	120.4	N7—Ru1—N6	90.09 (13)
C50—C51—H51	120.4	N7—Ru1—N1	96.51 (13)
N10—C52—C51	122.8 (4)	N6—Ru1—N1	171.77 (13)
N10—C52—H52	118.6	N7—Ru1—N5	96.86 (12)
C51—C52—H52	118.6	N6—Ru1—N5	78.30 (12)
N11—C53—C54	121.0 (4)	N1—Ru1—N5	95.98 (12)
N11—C53—C46	119.0 (3)	N7—Ru1—N2	173.88 (12)
C54—C53—C46	120.0 (4)	N6—Ru1—N2	94.25 (12)
N12—C54—C53	120.1 (3)	N1—Ru1—N2	79.56 (12)
N12—C54—C49	119.9 (3)	N5—Ru1—N2	88.26 (12)
C53—C54—C49	120.0 (4)	N7—Ru1—N8	78.14 (13)
N11—C55—C60	120.9 (3)	N6—Ru1—N8	94.46 (12)
N11—C55—C56	117.1 (3)	N1—Ru1—N8	91.71 (12)
C60—C55—C56	122.0 (3)	N5—Ru1—N8	171.29 (12)
C55—C56—C57	113.5 (3)	N2—Ru1—N8	97.19 (12)
C55—C56—H56A	108.9	N15—Ru2—N10	95.73 (13)
C57—C56—H56A	108.9	N15—Ru2—N14	95.98 (13)
C55—C56—H56B	108.9	N10—Ru2—N14	97.54 (13)
C57—C56—H56B	108.9	N15—Ru2—N16	78.30 (13)
H56A—C56—H56B	107.7	N10—Ru2—N16	169.02 (13)
C58—C57—C56	111.2 (3)	N14—Ru2—N16	92.28 (13)
C58—C57—H57A	109.4	N15—Ru2—N9	87.55 (13)
C56—C57—H57A	109.4	N10—Ru2—N9	79.34 (13)
C58—C57—H57B	109.4	N14—Ru2—N9	175.53 (12)
C56—C57—H57B	109.4	N16—Ru2—N9	91.12 (12)
H57A—C57—H57B	108.0	N15—Ru2—N13	172.03 (13)
C57—C58—C59	111.9 (4)	N10—Ru2—N13	90.41 (13)
C57—C58—H58A	109.2	N14—Ru2—N13	78.14 (12)
C59—C58—H58A	109.2	N16—Ru2—N13	96.43 (13)
C57—C58—H58B	109.2	N9—Ru2—N13	98.59 (12)
C59—C58—H58B	109.2		
			
N1—C1—C2—C3	−1.7 (6)	C4—C11—N3—C13	178.4 (3)
C1—C2—C3—C4	2.2 (6)	C13—C18—N4—C12	−1.8 (5)
C2—C3—C4—C5	−0.9 (5)	C17—C18—N4—C12	177.5 (3)
C2—C3—C4—C11	177.8 (4)	C11—C12—N4—C18	0.5 (5)
C11—C4—C5—N1	−179.7 (4)	C7—C12—N4—C18	−178.7 (3)
C3—C4—C5—N1	−0.9 (6)	C22—C23—N5—C19	3.9 (5)
C11—C4—C5—C6	0.0 (6)	C24—C23—N5—C19	−175.6 (3)
C3—C4—C5—C6	178.8 (3)	C22—C23—N5—Ru1	−170.0 (3)
N1—C5—C6—N2	0.8 (5)	C24—C23—N5—Ru1	10.5 (4)
C4—C5—C6—N2	−178.9 (3)	C20—C19—N5—C23	−2.1 (5)
N1—C5—C6—C7	179.7 (3)	C20—C19—N5—Ru1	171.0 (3)
C4—C5—C6—C7	0.0 (6)	C25—C24—N6—C28	1.4 (6)
N2—C6—C7—C12	178.8 (3)	C23—C24—N6—C28	−179.4 (3)
C5—C6—C7—C12	0.0 (5)	C25—C24—N6—Ru1	179.8 (3)
N2—C6—C7—C8	0.8 (5)	C23—C24—N6—Ru1	−1.0 (5)
C5—C6—C7—C8	−178.0 (3)	C27—C28—N6—C24	−1.0 (5)
C6—C7—C8—C9	−1.0 (5)	C27—C28—N6—Ru1	−179.3 (3)
C12—C7—C8—C9	−178.9 (3)	C32—C31—N7—C35	−4.3 (6)
C7—C8—C9—C10	0.6 (6)	C32—C31—N7—Ru1	171.8 (3)
C8—C9—C10—N2	0.1 (6)	C34—C35—N7—C31	5.9 (6)
C5—C4—C11—N3	179.2 (3)	C36—C35—N7—C31	−172.1 (3)
C3—C4—C11—N3	0.5 (6)	C34—C35—N7—Ru1	−170.5 (3)
C5—C4—C11—C12	0.0 (6)	C36—C35—N7—Ru1	11.5 (4)
C3—C4—C11—C12	−178.7 (4)	C39—C40—N8—C36	−1.3 (5)
N3—C11—C12—N4	1.7 (6)	C39—C40—N8—Ru1	−175.0 (3)
C4—C11—C12—N4	−179.2 (3)	C37—C36—N8—C40	2.5 (6)
N3—C11—C12—C7	−179.1 (3)	C35—C36—N8—C40	−179.4 (3)
C4—C11—C12—C7	0.0 (5)	C37—C36—N8—Ru1	176.8 (3)
C6—C7—C12—N4	179.2 (3)	C35—C36—N8—Ru1	−5.1 (4)
C8—C7—C12—N4	−2.9 (5)	C44—C43—N9—C47	−1.8 (5)
C6—C7—C12—C11	0.0 (5)	C44—C43—N9—Ru2	177.0 (3)
C8—C7—C12—C11	177.9 (3)	C46—C47—N9—C43	−0.2 (6)
N3—C13—C14—C15	−167.6 (3)	C48—C47—N9—C43	−177.1 (3)
C18—C13—C14—C15	10.6 (5)	C46—C47—N9—Ru2	−179.2 (3)
C13—C14—C15—C16	−41.4 (4)	C48—C47—N9—Ru2	3.9 (4)
C14—C15—C16—C17	60.2 (4)	C51—C52—N10—C48	1.6 (5)
C15—C16—C17—C18	−45.9 (4)	C51—C52—N10—Ru2	−179.2 (3)
N3—C13—C18—N4	1.0 (5)	C49—C48—N10—C52	−0.5 (6)
C14—C13—C18—N4	−177.2 (3)	C47—C48—N10—C52	178.6 (3)
N3—C13—C18—C17	−178.3 (3)	C49—C48—N10—Ru2	−179.9 (3)
C14—C13—C18—C17	3.6 (5)	C47—C48—N10—Ru2	−0.8 (4)
C16—C17—C18—N4	−164.9 (3)	C60—C55—N11—C53	−3.5 (5)
C16—C17—C18—C13	14.4 (5)	C56—C55—N11—C53	174.0 (3)
N5—C19—C20—C21	−1.6 (5)	C54—C53—N11—C55	1.9 (5)
C19—C20—C21—C22	3.5 (5)	C46—C53—N11—C55	−175.9 (3)
C19—C20—C21—C29	−175.4 (3)	C55—C60—N12—C54	−1.6 (5)
C20—C21—C22—C23	−1.9 (5)	C59—C60—N12—C54	−179.8 (3)
C29—C21—C22—C23	177.1 (3)	C53—C54—N12—C60	−0.1 (5)
C21—C22—C23—N5	−1.9 (6)	C49—C54—N12—C60	177.6 (3)
C21—C22—C23—C24	177.6 (4)	C62—C61—N13—C65	2.8 (5)
N5—C23—C24—N6	−6.3 (5)	C62—C61—N13—Ru2	−177.6 (3)
C22—C23—C24—N6	174.2 (4)	C64—C65—N13—C61	−3.2 (5)
N5—C23—C24—C25	172.9 (4)	C66—C65—N13—C61	174.8 (3)
C22—C23—C24—C25	−6.6 (6)	C64—C65—N13—Ru2	177.1 (3)
N6—C24—C25—C26	−2.4 (6)	C66—C65—N13—Ru2	−4.9 (4)
C23—C24—C25—C26	178.5 (4)	C67—C66—N14—C70	0.9 (6)
C24—C25—C26—C27	2.9 (5)	C65—C66—N14—C70	−176.8 (3)
C24—C25—C26—C30	−177.2 (4)	C67—C66—N14—Ru2	−177.2 (3)
C25—C26—C27—C28	−2.6 (5)	C65—C66—N14—Ru2	5.1 (4)
C30—C26—C27—C28	177.5 (3)	C69—C70—N14—C66	−0.7 (6)
C26—C27—C28—N6	1.7 (6)	C69—C70—N14—Ru2	177.2 (3)
N7—C31—C32—C33	−1.7 (6)	C79—C78—N15—C82	2.8 (5)
C31—C32—C33—C34	5.9 (6)	C77—C78—N15—C82	−176.9 (3)
C31—C32—C33—C41	−174.7 (3)	C79—C78—N15—Ru2	−173.4 (3)
C32—C33—C34—C35	−4.3 (6)	C77—C78—N15—Ru2	6.9 (4)
C41—C33—C34—C35	176.3 (4)	C81—C82—N15—C78	−2.3 (5)
C33—C34—C35—N7	−1.6 (6)	C81—C82—N15—Ru2	173.5 (3)
C33—C34—C35—C36	176.2 (4)	C76—C77—N16—C73	−2.0 (6)
N7—C35—C36—N8	−4.1 (5)	C78—C77—N16—C73	−179.4 (3)
C34—C35—C36—N8	178.0 (4)	C76—C77—N16—Ru2	170.6 (3)
N7—C35—C36—C37	173.9 (4)	C78—C77—N16—Ru2	−6.8 (4)
C34—C35—C36—C37	−4.0 (6)	C74—C73—N16—C77	1.1 (6)
N8—C36—C37—C38	−0.6 (6)	C74—C73—N16—Ru2	−170.7 (3)
C35—C36—C37—C38	−178.5 (4)	C31—N7—Ru1—N6	−92.5 (3)
C36—C37—C38—C39	−2.4 (6)	C35—N7—Ru1—N6	83.7 (3)
C36—C37—C38—C42	178.1 (4)	C31—N7—Ru1—N1	82.6 (3)
C37—C38—C39—C40	3.5 (5)	C35—N7—Ru1—N1	−101.2 (3)
C42—C38—C39—C40	−177.0 (4)	C31—N7—Ru1—N5	−14.2 (3)
C38—C39—C40—N8	−1.7 (5)	C35—N7—Ru1—N5	161.9 (3)
N9—C43—C44—C45	2.5 (6)	C31—N7—Ru1—N8	173.0 (3)
C43—C44—C45—C46	−1.3 (6)	C35—N7—Ru1—N8	−10.9 (3)
C44—C45—C46—C47	−0.5 (6)	C24—N6—Ru1—N7	102.0 (3)
C44—C45—C46—C53	177.6 (4)	C28—N6—Ru1—N7	−79.7 (3)
C53—C46—C47—N9	−176.8 (4)	C24—N6—Ru1—N5	5.0 (3)
C45—C46—C47—N9	1.3 (6)	C28—N6—Ru1—N5	−176.7 (3)
C53—C46—C47—C48	0.0 (6)	C24—N6—Ru1—N2	−82.3 (3)
C45—C46—C47—C48	178.1 (3)	C28—N6—Ru1—N2	96.0 (3)
N9—C47—C48—N10	−2.1 (5)	C24—N6—Ru1—N8	−179.9 (3)
C46—C47—C48—N10	−179.1 (3)	C28—N6—Ru1—N8	−1.6 (3)
N9—C47—C48—C49	177.0 (3)	C1—N1—Ru1—N7	−7.3 (3)
C46—C47—C48—C49	0.0 (6)	C5—N1—Ru1—N7	172.8 (3)
N10—C48—C49—C54	179.1 (3)	C1—N1—Ru1—N5	90.3 (3)
C47—C48—C49—C54	0.0 (6)	C5—N1—Ru1—N5	−89.6 (3)
N10—C48—C49—C50	−0.8 (5)	C1—N1—Ru1—N2	177.4 (3)
C47—C48—C49—C50	−179.9 (3)	C5—N1—Ru1—N2	−2.5 (3)
C54—C49—C50—C51	−178.7 (3)	C1—N1—Ru1—N8	−85.6 (3)
C48—C49—C50—C51	1.2 (5)	C5—N1—Ru1—N8	94.5 (3)
C49—C50—C51—C52	−0.2 (5)	C23—N5—Ru1—N7	−97.3 (3)
C50—C51—C52—N10	−1.2 (5)	C19—N5—Ru1—N7	89.5 (3)
C47—C46—C53—N11	177.8 (4)	C23—N5—Ru1—N6	−8.6 (3)
C45—C46—C53—N11	−0.2 (6)	C19—N5—Ru1—N6	178.2 (3)
C47—C46—C53—C54	0.0 (6)	C23—N5—Ru1—N1	165.4 (3)
C45—C46—C53—C54	−178.0 (4)	C19—N5—Ru1—N1	−7.8 (3)
N11—C53—C54—N12	0.0 (6)	C23—N5—Ru1—N2	86.1 (3)
C46—C53—C54—N12	177.7 (4)	C19—N5—Ru1—N2	−87.1 (3)
N11—C53—C54—C49	−177.7 (3)	C10—N2—Ru1—N6	−4.4 (3)
C46—C53—C54—C49	0.0 (6)	C6—N2—Ru1—N6	177.4 (2)
C48—C49—C54—N12	−177.8 (3)	C10—N2—Ru1—N1	−178.9 (3)
C50—C49—C54—N12	2.1 (6)	C6—N2—Ru1—N1	2.8 (2)
C48—C49—C54—C53	0.0 (6)	C10—N2—Ru1—N5	−82.5 (3)
C50—C49—C54—C53	179.9 (3)	C6—N2—Ru1—N5	99.2 (3)
N11—C55—C56—C57	168.8 (3)	C10—N2—Ru1—N8	90.6 (3)
C60—C55—C56—C57	−13.7 (5)	C6—N2—Ru1—N8	−87.6 (3)
C55—C56—C57—C58	41.8 (5)	C40—N8—Ru1—N7	−177.4 (3)
C56—C57—C58—C59	−62.0 (4)	C36—N8—Ru1—N7	8.6 (3)
C57—C58—C59—C60	51.1 (5)	C40—N8—Ru1—N6	93.4 (3)
N11—C55—C60—N12	3.5 (5)	C36—N8—Ru1—N6	−80.5 (3)
C56—C55—C60—N12	−173.9 (3)	C40—N8—Ru1—N1	−81.1 (3)
N11—C55—C60—C59	−178.3 (4)	C36—N8—Ru1—N1	104.9 (3)
C56—C55—C60—C59	4.3 (5)	C40—N8—Ru1—N2	−1.5 (3)
C58—C59—C60—N12	156.0 (3)	C36—N8—Ru1—N2	−175.4 (3)
C58—C59—C60—C55	−22.3 (5)	C78—N15—Ru2—N10	162.7 (3)
N13—C61—C62—C63	−0.4 (5)	C82—N15—Ru2—N10	−13.2 (3)
C61—C62—C63—C64	−1.5 (5)	C78—N15—Ru2—N14	−99.0 (3)
C61—C62—C63—C71	176.6 (3)	C82—N15—Ru2—N14	85.1 (3)
C62—C63—C64—C65	1.0 (5)	C78—N15—Ru2—N16	−7.9 (3)
C71—C63—C64—C65	−177.1 (3)	C82—N15—Ru2—N16	176.2 (3)
C63—C64—C65—N13	1.3 (5)	C78—N15—Ru2—N9	83.7 (3)
C63—C64—C65—C66	−176.5 (3)	C82—N15—Ru2—N9	−92.2 (3)
N13—C65—C66—N14	−0.1 (5)	C52—N10—Ru2—N15	96.5 (3)
C64—C65—C66—N14	177.9 (3)	C48—N10—Ru2—N15	−84.2 (3)
N13—C65—C66—C67	−177.8 (4)	C52—N10—Ru2—N14	−0.3 (3)
C64—C65—C66—C67	0.2 (6)	C48—N10—Ru2—N14	178.9 (3)
N14—C66—C67—C68	−1.5 (6)	C52—N10—Ru2—N16	152.9 (6)
C65—C66—C67—C68	176.1 (4)	C48—N10—Ru2—N16	−27.8 (8)
C66—C67—C68—C69	1.7 (6)	C52—N10—Ru2—N9	−177.1 (3)
C66—C67—C68—C72	−178.0 (4)	C48—N10—Ru2—N9	2.2 (3)
C67—C68—C69—C70	−1.5 (6)	C52—N10—Ru2—N13	−78.4 (3)
C72—C68—C69—C70	178.2 (4)	C48—N10—Ru2—N13	100.9 (3)
C68—C69—C70—N14	1.0 (6)	C66—N14—Ru2—N15	168.7 (3)
N16—C73—C74—C75	0.7 (6)	C70—N14—Ru2—N15	−9.2 (3)
C73—C74—C75—C76	−1.5 (6)	C66—N14—Ru2—N10	−94.7 (3)
C73—C74—C75—C83	177.7 (4)	C70—N14—Ru2—N10	87.4 (3)
C74—C75—C76—C77	0.6 (6)	C66—N14—Ru2—N16	90.2 (3)
C83—C75—C76—C77	−178.6 (4)	C70—N14—Ru2—N16	−87.7 (3)
C75—C76—C77—N16	1.2 (6)	C66—N14—Ru2—N13	−5.9 (3)
C75—C76—C77—C78	178.3 (4)	C70—N14—Ru2—N13	176.2 (3)
N16—C77—C78—N15	−0.1 (5)	C77—N16—Ru2—N15	8.0 (3)
C76—C77—C78—N15	−177.4 (4)	C73—N16—Ru2—N15	−180.0 (3)
N16—C77—C78—C79	−179.8 (4)	C77—N16—Ru2—N10	−49.8 (8)
C76—C77—C78—C79	2.9 (6)	C73—N16—Ru2—N10	122.2 (6)
N15—C78—C79—C80	−1.0 (6)	C77—N16—Ru2—N14	103.6 (3)
C77—C78—C79—C80	178.7 (4)	C73—N16—Ru2—N14	−84.3 (3)
C78—C79—C80—C81	−1.5 (6)	C77—N16—Ru2—N9	−79.3 (3)
C78—C79—C80—C84	177.1 (4)	C73—N16—Ru2—N9	92.8 (3)
C79—C80—C81—C82	2.0 (5)	C77—N16—Ru2—N13	−178.0 (3)
C84—C80—C81—C82	−176.5 (3)	C73—N16—Ru2—N13	−6.0 (3)
C80—C81—C82—N15	−0.2 (6)	C43—N9—Ru2—N15	−85.8 (3)
C2—C1—N1—C5	−0.2 (6)	C47—N9—Ru2—N15	93.1 (3)
C2—C1—N1—Ru1	180.0 (3)	C43—N9—Ru2—N10	177.9 (3)
C4—C5—N1—C1	1.5 (6)	C47—N9—Ru2—N10	−3.2 (3)
C6—C5—N1—C1	−178.2 (3)	C43—N9—Ru2—N16	−7.5 (3)
C4—C5—N1—Ru1	−178.6 (3)	C47—N9—Ru2—N16	171.3 (3)
C6—C5—N1—Ru1	1.7 (4)	C43—N9—Ru2—N13	89.1 (3)
C9—C10—N2—C6	−0.3 (6)	C47—N9—Ru2—N13	−92.0 (3)
C9—C10—N2—Ru1	−178.4 (3)	C61—N13—Ru2—N10	−76.3 (3)
C7—C6—N2—C10	−0.2 (5)	C65—N13—Ru2—N10	103.4 (3)
C5—C6—N2—C10	178.7 (3)	C61—N13—Ru2—N14	−173.9 (3)
C7—C6—N2—Ru1	178.2 (3)	C65—N13—Ru2—N14	5.8 (3)
C5—C6—N2—Ru1	−2.9 (4)	C61—N13—Ru2—N16	95.1 (3)
C18—C13—N3—C11	1.2 (5)	C65—N13—Ru2—N16	−85.2 (3)
C14—C13—N3—C11	179.4 (3)	C61—N13—Ru2—N9	3.0 (3)
C12—C11—N3—C13	−2.4 (5)	C65—N13—Ru2—N9	−177.3 (3)
